# Lab-on-PCB and Flow Driving: A Critical Review

**DOI:** 10.3390/mi12020175

**Published:** 2021-02-10

**Authors:** Francisco Perdigones

**Affiliations:** Electronic Engineering Department, Higher Technical School of Engineering, University of Seville, 41092 Seville, Spain; fperdigones@us.es

**Keywords:** lab-on-PCB, microfluidics, flow driving, actuators, biomedical applications

## Abstract

Lab-on-PCB devices have been developed for many biomedical and biochemical applications. However, much work has to be done towards commercial applications. Even so, the research on devices of this kind is rapidly increasing. The reason for this lies in the great potential of lab-on-PCB devices to provide marketable devices. This review describes the active flow driving methods for lab-on-PCB devices, while commenting on their main characteristics. Among others, the methods described are the typical external impulsion devices, that is, syringe or peristaltic pumps; pressurized microchambers for precise displacement of liquid samples; electrowetting on dielectrics; and electroosmotic and phase-change-based flow driving, to name a few. In general, there is not a perfect method because all of them have drawbacks. The main problems with regard to marketable devices are the complex fabrication processes, the integration of many materials, the sealing process, and the use of many facilities for the PCB-chips. The larger the numbers of integrated sensors and actuators in the PCB-chip, the more complex the fabrication. In addition, the flow driving-integrated devices increase that difficulty. Moreover, the biological applications are demanding. They require transparency, biocompatibility, and specific ambient conditions. All the problems have to be solved when trying to reach repetitiveness and reliability, for both the fabrication process and the working of the lab-on-PCB, including the flow driving system.

## 1. Introduction

Lab-on-PCB has been the subject of increasing research over the last few years [1,2]. These devices emerged as a promising evolution of lab-on-chip devices [3,4,5] and the PCB-MEMS technology [6]. They share important properties with lab-on-chip devices—for example, small fluid volume and rapid response time. Particularly, the core of these devices is the integration of sensors for measuring the results of a reaction, and for controlling the parameters of the samples; and the integration of actuators for conditioning the samples and for moving those samples through the microfluidic platform.

The need for micromixing, microheating, and sensing in different parts of the microfluidic platforms makes the control of liquids mandatory. For this reason, the flow driving and fluid manipulation into a network of microchannels is one of the most important issues for lab-on-chip devices (LoC) and platforms [3,7], and particularly for lab-on-PCB. In this respect, the first attempts of developing a fluid manipulation date to late last century with a gas chromatographic air analyzer, and a miniaturized electrophoresis system [8,9]. Many works about flow driving have been carried out since those years, providing a large number of methods for performing similar tasks. Several of these methods have been integrated in lab-on-PCB: among others, pressurized microchambers, peristaltic pumps, and electrowetting on dielectrics.

Although lab-on-chip and lab-on-PCB have characteristics in common, and lab-on-PCB can be considered as a kind of lab-on-chip, they are different platforms. For example, unlike lab-on-chips, lab-on-PCBs are interesting due to the easy integration of microfluidics and electronics in the same platform, towards self-contained systems for microfluidic applications [10,11,12]. Apart from the integration, the interest in lab-on-PCB devices lies in the commercial availability of the PCB substrate with very reasonable dimensions at low cost [13,14]. Thanks to this characteristic, the lab-on-PCB devices can be disposable at low cost. This is important because the cleaning cost can be avoided. In fact, the cleaning implies an auxiliary and integrated microfluidic circuit or the use of the external facilities. The first option means an increase of the chip area, and thus a higher price. The cost of the second option is not worth worrying about because the devices are inexpensive. Moreover, the cleaning of small microchannels is very demanding, especially in biomedical applications. Therefore, the best choice to avoid cross-contamination from the biological and economical point of view is the use of freshly fabricated devices. As previously said, lab-on-PCB devices are a very interesting option due to their low cost. This is an important difference with respect to lab-on-chip devices, and makes the lab-on-PCB devices an attractive choice from the market point of view. However, these single-use devices imply environmental issues due to the metals of the PCBs. Fortunately, the electronics industry has been using PCBs for over 50 years and this issue is solved.

The main differentiating characteristics of lab-on-chip and lab-on-PCB are summarized in Table 1.

**Figure 1:**
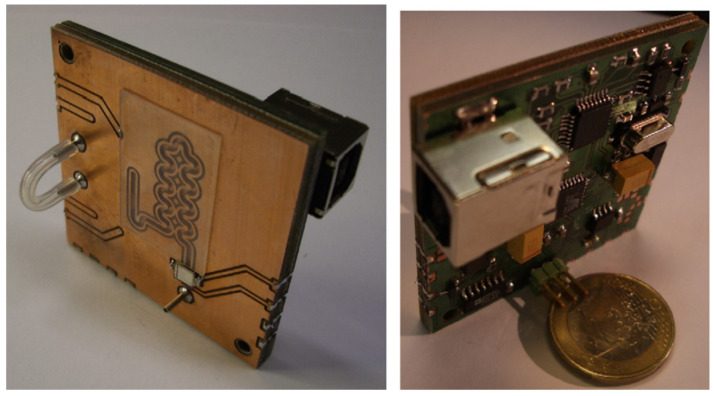
The first lab-on-PCB reported by Stefan Gassmann et al. (Reprinted from [20], copyright (2007), with permission from Elsevier).

As can be seen, the lab-on-PCB devices could be entirely fabricated using printed circuit boards; however, the lack of transparency makes them not very useful for optical measurement systems. In order to solve this issue, the PCB has been integrated with transparent materials, for example, glass, SU-8, PDMS, or kapton. For instance, the first platform integrating electronics and microfluidics using printed circuit boards (PCB) was developed by Jobst et al. [16] at the Technical University of Vienna in 1997, for the fabrication of a microdevice monitoring different metabolites by a biosensor array fabricated using glass. Two years later, Pagel and coworkers at the University of Rostock laid out the basis of the PCB technology, and they used it for developing several PCB-based devices, also named PCBMEMS devices [17,18,19]. The first lab-on-PCB itself also included a transparent cover. It was reported by Stefan Gassmann et al. [20] in 2007; see Figure 1.

This device was composed of a microfluidic platform with integrated electronics, sensors, actuators, a transparent cover, and fluid manipulation for the detection of $Fe^{3+}$—that is, a lab-on-PCB with all its possible components and a specific application.

Glass, SU-8, PDMS, and kapton are very useful for fabricating prototypes, but there are better options with which to develop a commercial product. In this respect, an industrial lab-on-PCB device requires rapid mass production; that is, the fabrication of that product has to be performed at as low a cost as possible, while generating the largest number of products at the same time. For this reason, the thermoplastic materials are a good choice [21]. Most of them are transparent with a well-established mass production procedure, such as injection molding or hot embossing. These materials and fabrication methods should be chosen to fabricate a highly integrable flow driving systems, in order to develop marketable lab-on-PCB chips.

The final target in the development of lab-on-PCB devices lies on the mass production of commercial products. In fact, the fluid manipulation together with the PCB technology are very important from the point of view of the market, because they make it possible to tackle the development of inexpensive devices for many different biomedical and chemical analyses.

Nowadays, there is much work to do about the control of fluids and their integration into lab-on-PCB devices. Despite the improvements developed in recent years, lab-on-PCB is far from being robust. Unlike microelectronic chips, lab-on-PCB devices require a highly multidisciplinary R&D group, and they have a lack of standardization for both design and end-user interfaces.

Regarding the future outlook, the authors suggest the reading of [1], especially the Section 4, where a complete analysis of the future is performed.

**Figure 2:**
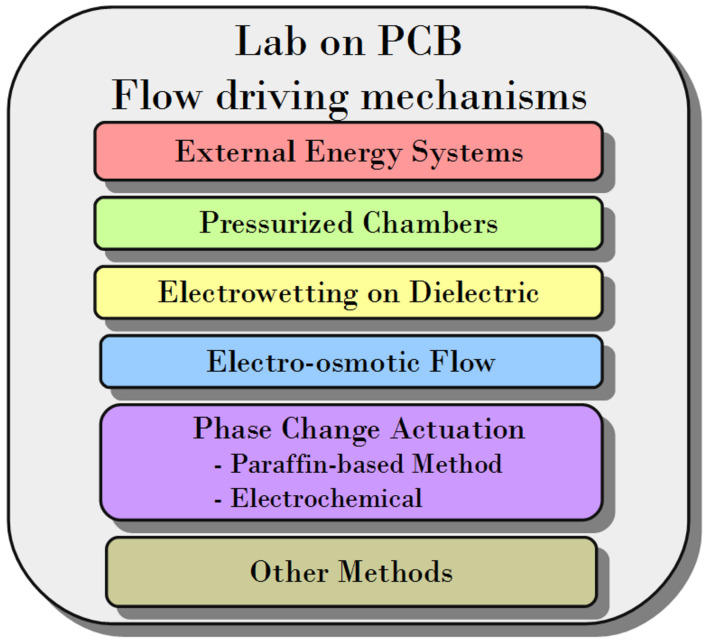
The external impulsion method together with the alternative ones mentioned in this review.

Historically, the most used flow driving mechanisms in lab-on-chips, and especially in lab-on-PCBs are external pressure sources and syringe pumps. This is a well-established method, but reduces the portability of the whole system extremely. However, this method does not necessarily imply a disadvantage, as will be explained in the discussion section. The tendency to reduce or completely remove the connection between the microfluidic platform and the external sources is very positive from the portability point of view. Apart from the handling of fluids, the portability is hugely related to the marketable products. The reason for that lies in the commercial potential of the point of care devices, and the launch of new products into the market. The lab-on-PCB mechanisms mentioned in this review can seen in Figure 2.

## 2. External Energy Systems

**Figure 3:**
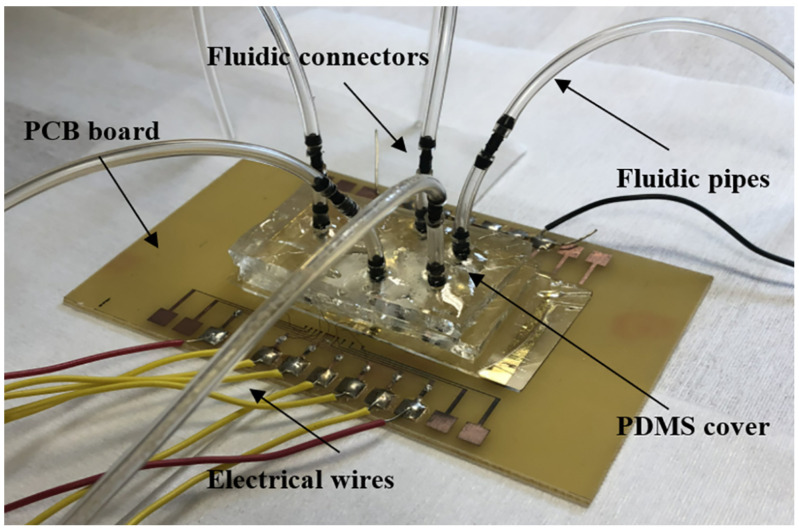
Prototype of a PCB-based biosensor for rapid detection of *Salmonella* in food products. The device requires the connection of five tubes, Ref. [24].

One of the most common methods to impulse liquids inside lab-on-chips and lab-on-PCBs consists of using syringe pumps. This method implies the use of tubing to connect these energy sources with the microfluidic platforms. In general, the number of pressure sources is a function of the number of liquids. This could be excessive for large-scale integration in microfluidics [22,23]. For example, a prototype of a PCB-based biosensor for rapid detection of *Salmonella* in food products [24] is shown in Figure 3. As can be seen, five tubes are connected to the device.

There are systems for multi-impulsion of liquids or gases to solve this problem, with the subsequent increase of cost. In addition, the connection of the tubes is an important issue to be solved. In this respect, multichannel chip-to-world interfaces for plug and play have been reported [25,26,27]. This solution consists of defining ports in microfluidics which tackle the required standardization. However, the portability continues to be an issue to face.

Many lab-on-PCB devices use external energy sources, such as syringe pumps, for moving the liquids with continuous flow. Although several combinations of external energy sources with lab-on-PCBs have been reported in the past [16,28,29,30], the examples chosen for this mechanism are a representative group of the recent past and present. For instance, the lab-on-PCB device reported by Moschou et al. [31] (2015) demonstrated the integration of stable Ag/AgCl pseudo-reference electrodes. In order to do so, the authors used a laboratory syringe pump (Chemyx Inc., Fusion 200, Stafford, TX, USA) for moving a buffer continuously through the reference electrode over 24 h. In 2017, a lab-on-PCB-based cytometer for detecting circulating tumor cells and enumeration was developed [32]. In this work, the biological samples were driven by a syringe pump (PHD 22/2000, Harvard Apparatus, MA, USA).

The PCB-based microfluidic platform for electrochemical detection of cancer biomarkers reported in [33] (2016) and the lab-on-PCB for conditioning the medium of cell cultures and mixing fluids described in [34] (2017), also require a connection to a syringe pump.

**Figure 4:**
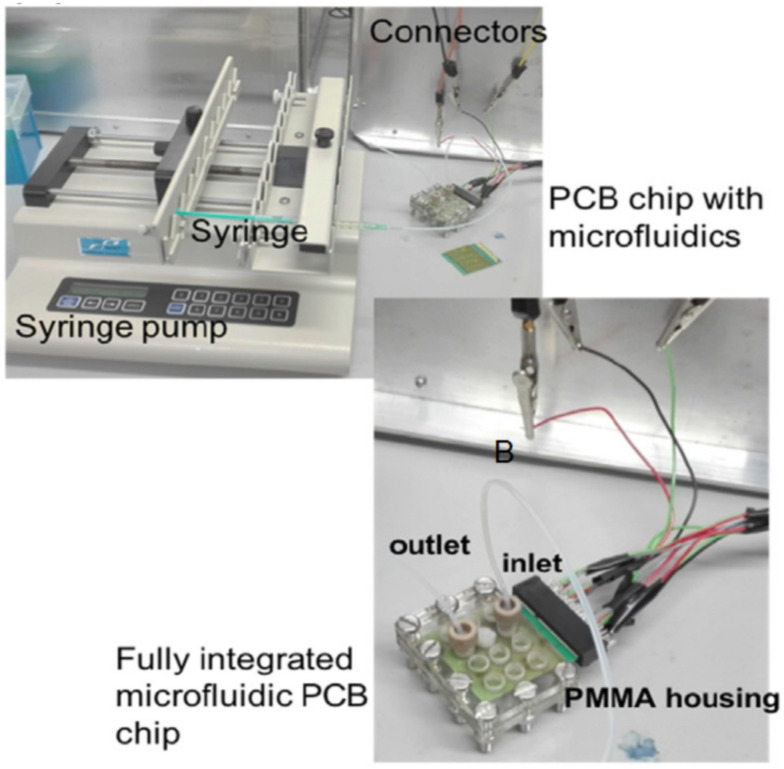
Lab-on-PCB for rapid and high sensitivity DNA quantification. The experiments are performed using an external syringe pump (Reprinted from [37], copyright (2019), with permission from Elsevier).

Recently, the PCB-based thermocycler for PCR developed in [35] (2019) required a syringe pump in order to move the DNA sample through a microchannel; the lab-on-PCB reported in [36] (2019) for organotypic cultures required continuous flow. Therefore, it needed to be connected to a external source (New Era Pump Systems, Inc, Farmingdale, NY, USA) to feed the tissues with culture medium. In addition, the lab-on-PCB for rapid and high sensitivity DNA quantification reported in [37] (2019) also made use of a syringe pump (Cole Palmer 230-CE) for continuous flow experiments; the reagents were delivered into the lab-on-PCB inlet; see Figure 4. The same syringe was used for delivering glucose samples in a lab-on-PCB for electrochemical glucose sensing (2020) [38]. Finally, peristaltic micropumps have also been used as external impulsion for lab-on-PCBs [39].

As can be seen, the external sources have been used for moving liquids in lab-on-PCBs from the beginning of this kind of device until today. This fact shows that this method continues being a good alternative to developing new devices with new applications. Although the external syringe pumps reduce the portability of the system, they are an interesting choice to demonstrate the integration of sensors and actuators in lab-on-PCB. Apart from this, many studies has been performed in order to remove the connection tubes from the microfluidic devices, aiming for portability.

## 3. Pressurized Chambers

The method based on pressurized microchambers [40,41] is a good alternative for moving liquids in lab-on-PCBs. This is a mechanism which allows the storage of pneumatic energy in a microchamber of the lab-on-PCB, so that the connection tubes can be avoided. Once the energy is available, it can be released by the opening of a microvalve. The activation of those microvalves is electrically performed. In order to do so, a gold wire with a diameter of 25 μm is used as a microheater.

**Figure 5:**
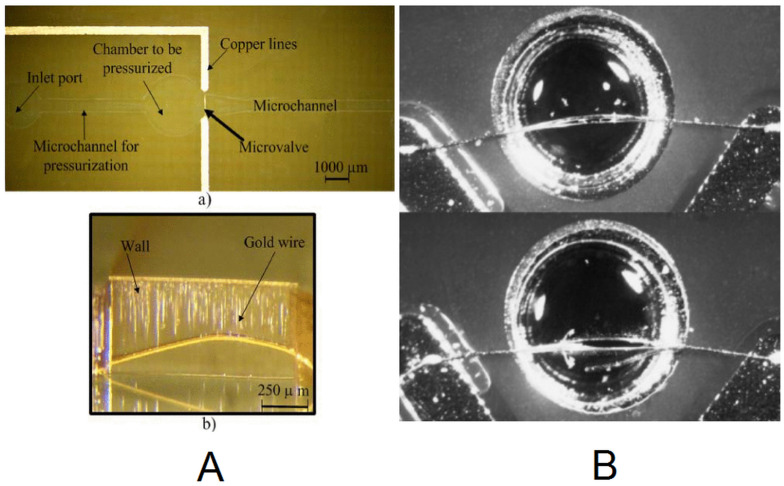
(**A**) Pressurized microchamber with vertical walls and an embedded gold wire (copyright (2014) IEEE. Reprinted, with permission, from [44]). (**B**) Pressurized microchamber composed of a planar SU-8 membrane with a completely embedded gold wire (Reprinted from [43], copyright (2010), with permission from Elsevier).

The pneumatic energy is stored inside SU-8 microchambers as high pressure air. The releasing of the air is achieved by the destruction of a thin vertical wall due to both the increase of temperature and the pressure of the microchamber; see Figure 5A. The gold wire is perpendicular to the wall and so it is not optimal. In this respect, several microvalves with the wire completely embedded in the wall have been reported [42,43,44]. These devices have vertical walls except the ones described in [43,45], where the SU-8 wall was a membrane transferred to the PCB substrate; see Figure 5B.

**Figure 6:**
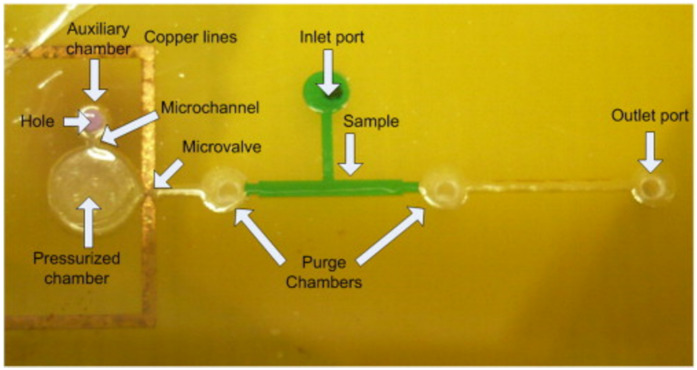
Impulsion system based on an SU-8 pressurized chamber and a copper line fuse. (Reprinted from [40], copyright (2015), with permission from Elsevier).

These microvalves were improved by using thin copper lines instead of wires [40,46]. They were fabricated by wet etching at the same time as the copper electronics tracks. This improvement implies the removing of the wire bonding step, so that the gold and the facilities involved to perform the bonding are avoided. In this case, the microvalve is used as a fuse, in order to destroy the SU-8 wall. An example of these devices is shown in Figure 6.

Several lab-on-PCB devices have been fabricated using this method—for example, the lab-on-PCB micromixer reported in [47], and the prototype developed to demonstrate the integration of a protocol in a lab-on-PCB platform [48].

**Figure 7:**
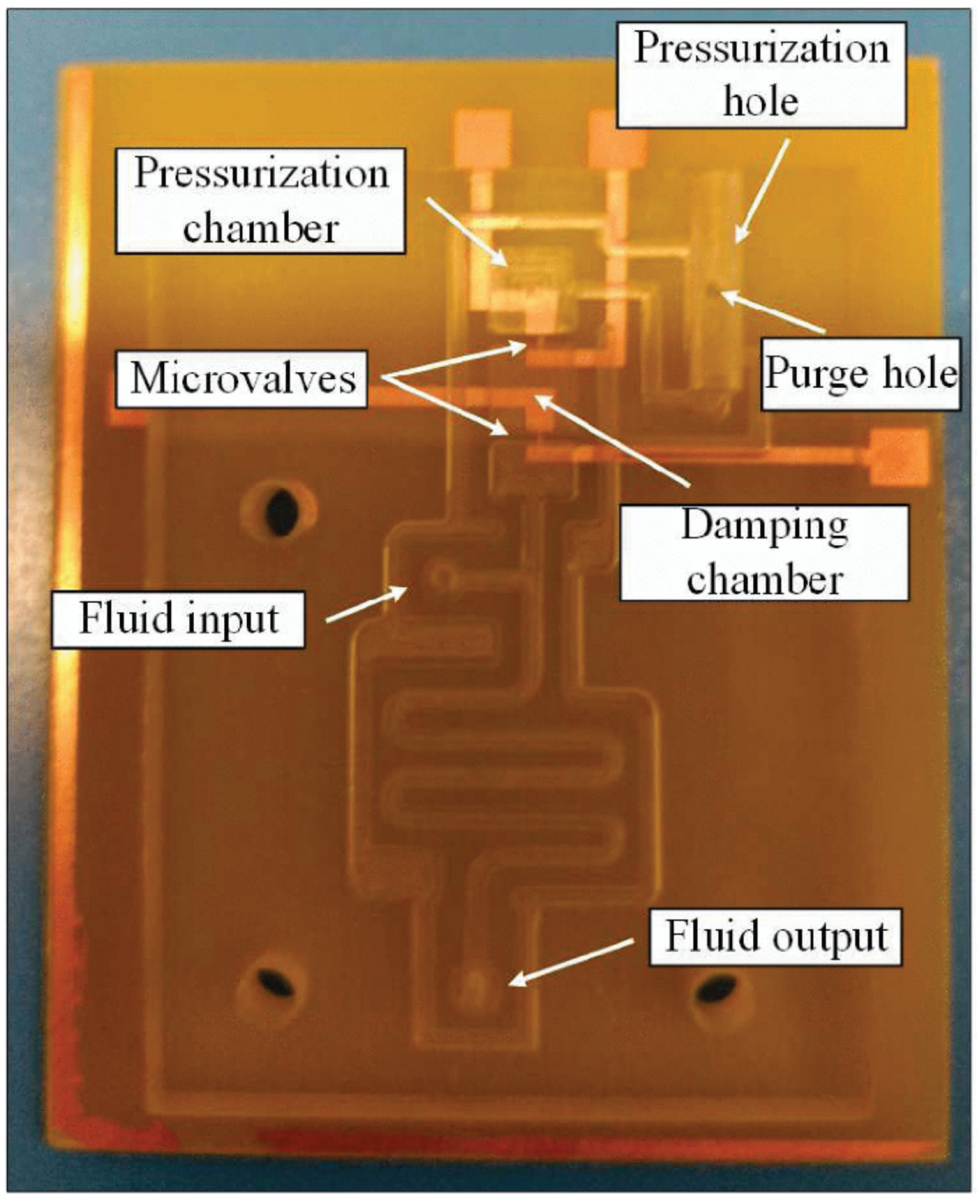
Pressurized chamber fabricated using a thermoplastic and PCB, with copper lines as a fuse [49]. (Copyright (2018) IEEE. Reprinted, with permission, from [49]).

Lab-on-PCBs have been fabricated using a thermoplastic (PMMA) and PCB, that is, materials compatible with mass production and multiple processes; see Figure 7. This device is closer to a marketable product than those using SU-8-based materials. Actually, the device shown in Figure 7 is fabricated via a computer numerical control (CNC) machine. This is not a mass production procedure, but the PMMA can be processed by injection molding or hot embossing.

In this case, the air has to be inserted in the microchamber using plungers [49]. The destruction of the fuse opens a small microchannel through which the air leaves the microchamber to impulse the liquid stored in the microfluidic circuit.

This method is mechanically simple due to the impulsion being based on no moving parts. With the electric energy required to destroy the copper fuse, the control and signal processing can be included in a unique electronic circuit.

Regarding the limitations of the method, it is important to comment on the lack of biocompatibility due to the destruction of the copper fuse. The debris of the copper lines could damage the biological samples. The authors propose an inert and intermediate liquid between the pressurized microchamber and the rest of the microfluidic circuit, so that the samples will never be in contact with the contaminated air. Nevertheless, the integration of the inert liquid reduces the simplicity of the whole system. In addition, it increases the complexity of fabrication. These facts imply an increase of the fabrication cost as a commercial product.

Finally, the gas permeability of the polymeric wall is important because the pressurized microchamber could discharge in the long-term. This is a problem for long-term storage as a product. Moreover, the handling of fluids using this method is limited to small samples. Continuous flow is not possible to achieve; for instance, the insertion of medium in a long-term cell culture cannot be managed by pressurized chambers. This is not a problem because this method is not intended to do so. There are better choices to those applications, for example, external energy sources.

## 4. Electrowetting on Dielectric

Electrowetting on dielectrics (EWOD) is one of the typical techniques of digital microfluidics [50,51,52]. This is a method whereby an electric field changes the wetting of a droplet, in contact with insulated and hydrophobic electrodes. The droplets are placed between two parallel layers; the bottom one is the substrate, which includes an electrode array covered with thin dielectric layer, and the top layer could be either a passive top plate or a ground plate. This method consists of switching the voltage to electrodes, so that the surface tension gradient can be modified, generating asymmetric contact angles, and the subsequent driving forces. Thus, the droplet is moved in two dimensions.

Among others, the advantages of EWOD devices include flexible device geometry, compatibility with other technologies, and simple electronic instrumentation. In addition, this technique allows easy manipulation of several reagents at a time, with a reduction of reagent volume, a short analysis time, and high sensitivity. Despite these advantages, the biomolecular adsorption of biological material due to the hydrophobic layer, together with electrolysis and evaporation of the small volume of liquids, and cross-contamination are the drawbacks of the technique, to name a few.

The PCB technology provides several advantages to EWOD devices. The typical fabrication process of these devices implies expensive clean rooms when processing glass substrates, resulting in smooth surfaces with reliable motion of droplets at voltages below 100 V. Integrated circuits offer the smooth surface topography required in the droplet manipulation. However, the PCB substrates provide low cost fabrication and quick turnaround time with slightly higher driven voltages [53].

**Figure 8:**
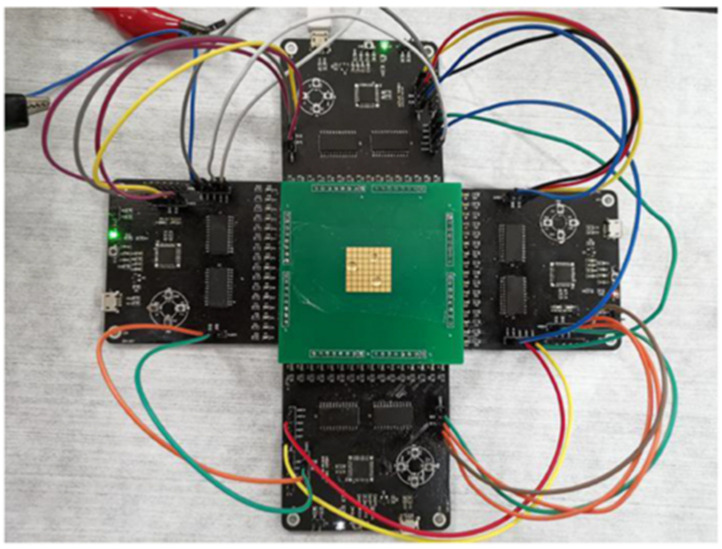
The open EWOD platform and the electronic circuit to control the droplets can be seen [57].

Many studies have taken advantage of the up-sides. For instance, the platform reported in [54] demonstrates the combination of lab-on-PCB and EWOD for two-plate and one-plane devices. The two-plate fabricated devices were used for moving, dispensing, merging, and splitting droplets in the range of 150–300 nL. On the other hand, the one-plate devices managed droplets with volumes of up to 3 μL. In addition, Parylene-C and PDMS were used as dielectric layers and the voltages ranged between 300 and 500 V at 18 kHz. Similar platforms have been reported for biological application and lower driven voltages, about AC voltage of 200 V at 1 kHz, for moving droplets of about 30 μL [55]. Besides, the works reported in [56] moved droplets of about 10 μL with a speed of 3 mm/s by applying a high DC voltage, 400 V. This very low cost device is composed of a planar array of six electrodes with silicone rubber as the dielectric layer and a commercially available water repellent as the hydrophobic layer. A similar open platform was reported in [57]; a planar array of 64 electrodes was developed. In this work, silicone oil and Parafilm M were used as a dielectric hydrophobic layer. The speed was 15 mm/s for droplets with a volume of 1850 μL, using a voltage output frequency of 10 Hz. This device is shown in Figure 8, where the open EWOD platform and the electronic circuit to control the droplets can be seen. Unlike the previously mentioned devices, the one reported in [58] was a two plate system with an array of 24 × 24 electrodes controlled by seven control signals at 15–30 V. It was composed of a dielectric layer (SU-8) and a hydrophobic layer (Teflon AF1600) deposited on the PCB.

The work described in [53] scales the PCB-based electrowetting devices to larger arrays, with smooth surface topography if taking into account the roughness of the PCB substrate. The authors state that they successfully moved, merged, and mixed droplet with volumes from 2 to 1200 μL. On the other hand, multilayer PCB substrates have also been used for EWOD [59,60,61]. Finally, PCB has also been used for contactless electrowetting, that is, modifying the contact angle by air ionization [62].

**Figure 9:**
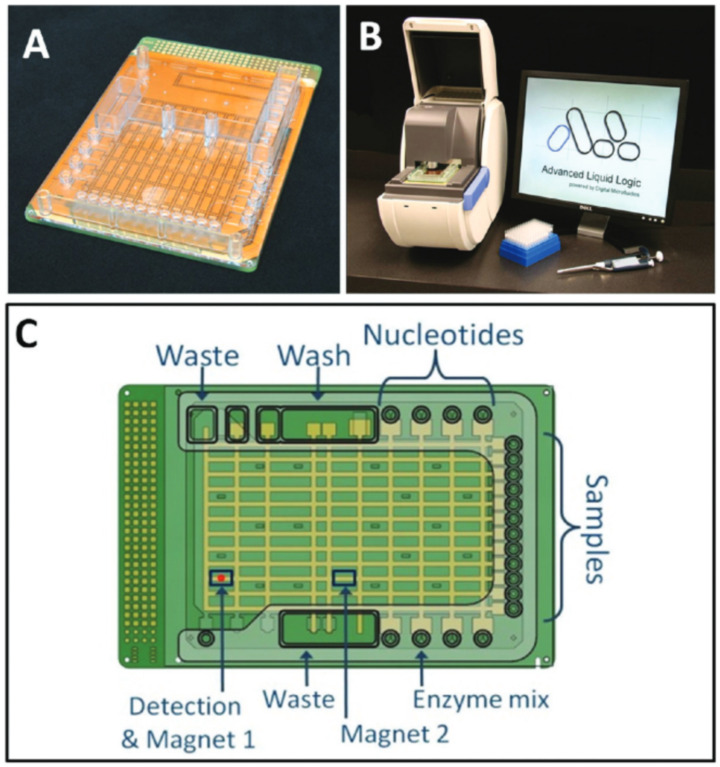
(**A**) Assembled multiwell-plate-sized PCB-based cartridge. (**B**) Photograph of the control instrument. and (**C**) Sketch of the cartridge showing the locations of sample and reagent wells (Reprinted with permission from [63], copyright (2011), American Chemical Society).

Apart from these prototypes and studies, the development towards a commercial product reported in [63,64] proposes droplet-based pyrosequencing and point of care devices as lab-on-PCB; see Figure 9.

In particular, the device is intended to perform both immunoassays for cardiac troponin I and real-time PCR assays with 300 nL droplets. Besides, the fabrication process is performed by utilizing mass production techniques.

As can be seen, lab-on-PCB devices based on electrowetting on dielectrics are a very interesting choice for many biological applications [65]. The technology is mature enough to stimulate the creation of companies based on PCB for biomedical applications [66].

In general, the problem with this kind of platform is related with the number of electrodes and the electronic circuit; that is, a large number of electrodes implies a larger number of electronic components in the circuit. Even so, this fact does not seem to be a drawback for industrial production. Regarding the fabrication process, it only requires the commercially available printed circuit board process and several coatings, together with a plastic part to store liquids. Finally, it is worth mentioning that this method does not necessarily require microchannels, and thus the related fabrication processes.

## 5. Electroosmotic Flow

This kind of pump is based on an electrokinetic phenomenon (electroosmosis) [52,67,68]. This process is used for impulsion liquids through microchannels or porous media, via the application of an external electric field. The electroosmotic effect takes place when fluids containing polar molecules are in contact with solid surfaces, so that electric charges appear in those surfaces which are in contact with the liquids. Those charges are negative when the working fluid is water in contact with an insulating solid. In addition, positive charges are induced at the same time in a thin layer of the water very close to the surface, in order to keep the electric neutrality. The layers of electrical charges of the fluid are named “Helmholtz layers”, and the combination of these positive and negative layers is called the “electrical double layer”. When an electrical field is applied inside the microchannel, forces appear on the positive layer and the charges are moved towards the negative electrode, transporting the liquid with them. The majority of electroosmotic pumps worked under direct current voltage. However, bubbles could appear at the driven electrodes due to the electrolysis. In this respect, the use of low alternating voltage can reduce or even eliminate the generation of bubbles.

Among other things, the main advantage of electroosmotic pumps is the generation pulse-free flows. In addition, the flow magnitude and direction can change instantly. Finally, like the impulsion systems based on pressurized chambers, electroosmotic pumps have no moving parts. Regarding the limitations, electrolysis and the bubble generation could appear at the metal electrodes. Moreover, the flow rates are low if compared to other integrated micropumps, and the microchannels required to achieved a given flow rate are relatively narrow.

Printed circuit boards are a good candidate to develop electroosmotic pumps due to their metal layer. This layer is used for fabricating the driven electrodes with the typical photolithographic process. Consequently, the integration can be performed with ease. The rest of the electroosmotic pump has to be fabricated with a different material.

**Figure 10:**
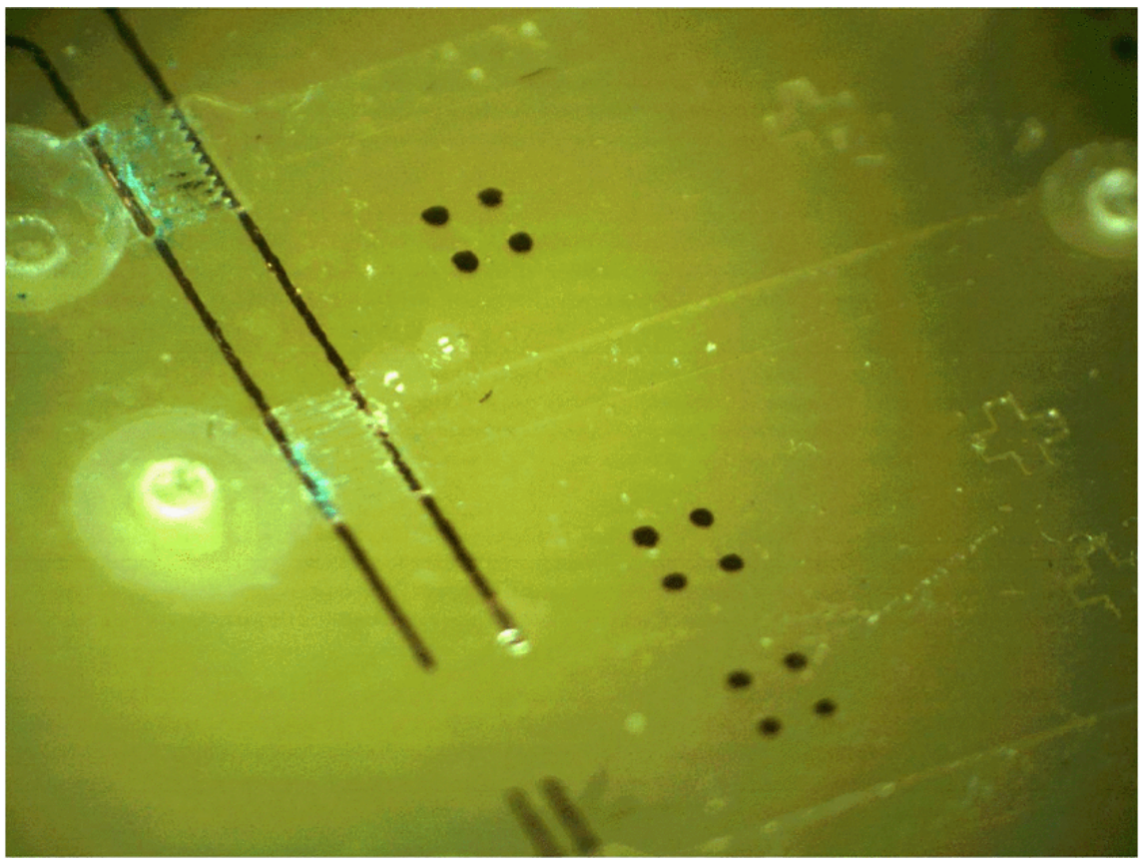
Prototype of an electroosmotic micropump for lab-on-PCBs. (Copyright (2012) IEEE. Reprinted, with permission, from [70]).

Several electroosmotic pumps have been integrated on PCBs—for example, the prototype reported in [69], which was fabricated using a PCB and SU-8; the Flame Retardant 4 (FR4) was the substrate, SU-8 was the material chosen with which to fabricate the microchannels and microchambers, and the copper layer was used for building the electrodes, electrical connections, and pads. The device is inexpensive but the materials make the mass production difficult. A similar pump with the same fabrication process was reported in [70]; see Figure 10. This pump is able to provide a flow rate of 1 μL/min at a direct current voltage of 60 V.

A different material was integrated with the PCB in a study presented in [71]; the authors describe a lab-on-PCB compatible with this flow driving method. The fabrication materials include a dry resist and PCB, and require hot pressing and several photolitographic processes. In addition, it includes surface mounted electronic components (SMD) intended to take measurements; for example, the authors performed optical experiments via the integration of two embedded SMD blue LEDs. They also integrated a temperature sensor and a resistor.

**Figure 11:**
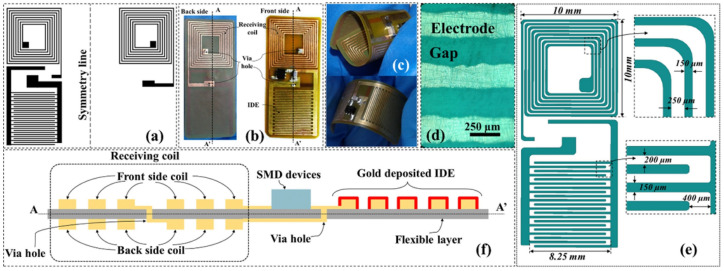
(**a**) Layout used for the device fabrication, (**b**) fabricated biased-AC electroosmotic lab-on-a-film pad, (**c**) demonstration of the device’s flexibility, (**d**) detail of the metal traces, (**e**) dimensions of the device pad, (**f**) cross-sectional view of the device along AA’ cut (Reprinted from [72], copyright (2016), with permission from Elsevier).

The work reported in [72] shows the design and characterization of a passive, disposable wireless for lab-on-PCB for particle and fluid manipulation. Unlike the previously mentioned devices, this one was fabricated on a flexible PCB (lab-on-a-film) integrating a receiving coil, an array of interdigitated electrodes (IDE), and two SMD components, a diode and a capacitor. It works at low voltages, and can perform three microfluidic operations depending on the wirelessly-controlled voltage, so that when the signal over an array of interdigitated electrodes is about 0.7 V, the IDE performs particle enrichment. The IDE works as an active mixer at 2 V; and as an AC electroosmotic pump when the voltage is 3 V. All of these functions are performed by the device itself with overall dimensions of 10 × 20 mm${}^{2}$; see Figure 11.

Finally, the micropump reported in [73] can be surface mounted on a commercial PCB. This pump is composed of a nanoporous membrane to provide an electroosmotic flow for a maximum flow rate of 8 μL/s at a voltage of 2 V.

Summarizing, electroosmotic pumps have been integrated into lab-on-PCBs for fluid manipulation. They provide a low cost choice for electrically controlled pulse-free continuous flows compatible with the integration of sensor and actuators. Nevertheless, external or integrated liquid reservoirs and narrow microchannels are required. In addition, electrolysis and bubbles could appear with inappropriate activation. On the other hand, the working of these devices with copper electrodes implies oxidation and the subsequent contamination of the fluid, and eventually the blocking of the microchannels (see the electrodes of the device of Figure 10). In order to avoid the oxidation, additional fabrication steps are required so that the fabrication cost increases due to both new facilities and new fabrication materials. In addition, the narrow microchannels are difficult to fabricate with mass production equipment with reasonable tolerance at low cost. This means a drawback from the industrial point of view.

## 6. Phase Change Actuation

The flow driving systems based on phase changes, such as microvalves and micropumps, have been used for microfluidic handling. The main methods for this purpose are based on paraffin [74] and electrochemical micropumps.

The paraffin-based method makes use of the thermal properties of the paraffin to change its phase from solid to liquid and vice versa. This material modifies its volume as a function of the temperature so that the higher the temperature is, the more the paraffin increases its volume, typically about 10–15%. This increase of volume and the melting are used for opening and closing valves in order to modify a flow rate. These systems have been included in lab-on-PCB platforms due to their advantages, such as simple design, and more importantly, the low cost of the paraffin. In addition, the paraffin-based valves can be activated several times, and they support high pressures [75]. However, these devices have several drawbacks, for example, the integration of the paraffin. Moreover, the characteristic of the material makes the complete system temperature-dependent. Finally, the time response to manipulate the liquids is high, about tens of seconds.

The increase of temperature required to change the phase of the paraffin has to be performed using a thermal actuator, that is, a microheater. Thus, the copper layer of the PCB substrate is very suitable to fabricate that microheater. It is developed as a serpentine copper line whose heating is governed by the Joule effect. This fact makes the paraffin-based actuation method easy to integrate in lab-on-PCB.

The paraffin-based method for fluid manipulation has been integrated in several lab-on-PCBs. Bodén et al. [76], in 2008, developed an on-chip liquid storage and dispensing for lab-on-PCB. The system has three integrated liquid reservoirs and dispenser units for 10 μL sample volumes. Those reservoirs can store either reagents or liquids for buffering or rinsing. The device is composed of a PCB substrate with an integrated copper microheater and an epoxy structure for the microfluidic circuit. Once the microheater is activated, the paraffin melts and expands, increasing the pressure. Therefore, the fluid is driven toward the microchambers and microchannels.

Recently, in 2019, Wang et al. [77] reported an on-board control of paraffin-based microfluidics manipulation on an active centrifugal lab-on-PCB for plasmid DNA extraction. The impulsion is based on both centrifugal forces and heating. The device is composed of a plastic microfluidic layer fabricated using a PMMA master, and a commercial PCB layer as a substrate. The heating is achieved by resistors in a PCB substrate. Unlike the previously mentioned paraffin-based lab-on-PCBs, the resistors are not serpentine-shaped copper lines. In this case, the temperature is raised using SMD resistors as thermal actuators. This implies a lower area of heating compared with the copper lines. Once the paraffin is melted, the centrifugal forces drive the fluid.

**Figure 12:**
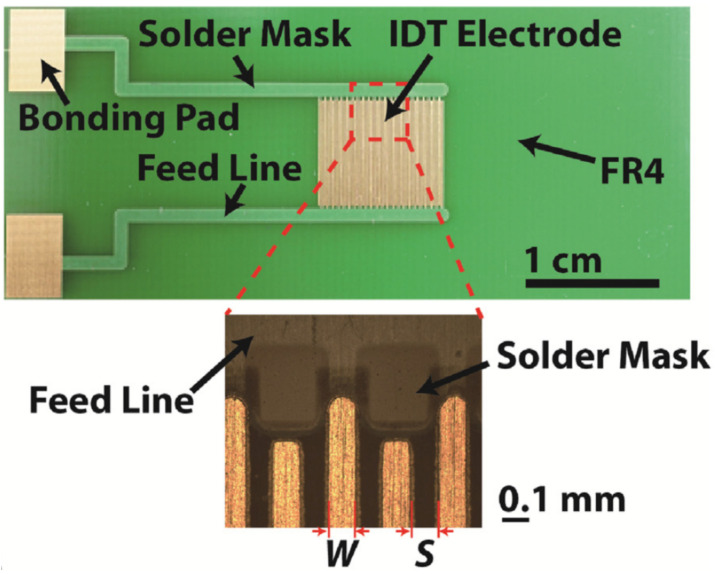
Electrochemical PCB-based impulsion chip with enlarged detail of the microelectrode fingers (Reprinted from [78], copyright (2018), with permission from Elsevier).

Regarding the electrochemical method, the most representative one is based on electrolysis, that is, the gas generation inside a liquid. Typically, the chosen liquid is water because it is inexpensive and easy to work with, and the resulting gases are hydrogen and oxygen. This method was applied to a PCB-based micropump with the maximum flow rate of 31.6 mL/min and maximum backpressure of 547 kPa (at 34 μL/min) [78,79]. The water electrolysis is achieved using integrated interdigitated microelectrodes on the PCB, where the metal of the electrodes is fabricated using the copper layer of the PCB with electroplated gold; see Figure 12.

A similar method was reported in [80] with a flow rate ranging from 20 μL/min to 135 μL/min. This device also uses integrated metal electrodes to perform the electrolysis of water. However, the electrodes are made of copper without electroplated gold. The very well-finished lab-on-PCB reported in [81] also uses the electrolytic gas generation inside a hydrogel. These electrolytic and PCB-based impulsion systems require the precise integration of a material inside the system, especially the last one, where the hydrogel has to be dispensed into the microchambers. Finally, the electrolytic method was applied for flow driving in a USB-based lab-on-PCB device for microparticle generation [82].

**Figure 13:**
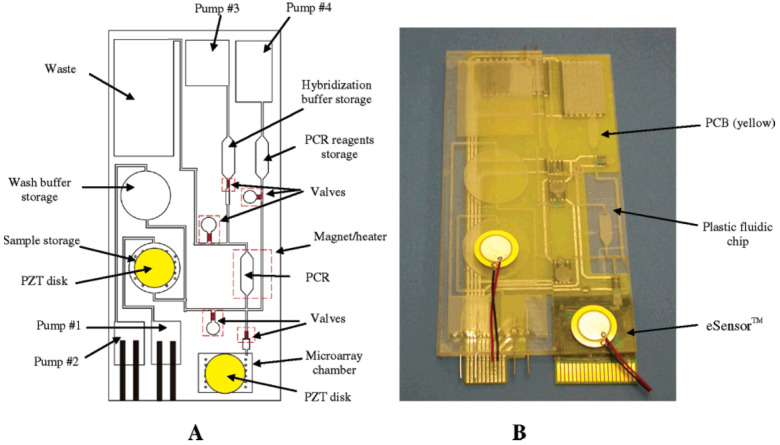
Self-contained, fully integrated lab-on-PCB for sample preparation, PCR amplification, and DNA detection. (**A**) Sketch of the plastic fluidic lab-on-PCB. Micropumps 1–3 are electrochemical, and pump 4 is thermopneumatic. (**B**) The integrated lab-on-PCB consists of a plastic microfluidic chip, a printed circuit board (PCB) substrate, and a Motorola eSensor microarray chip (Reprinted with permission from [11], copyright (2004), American Chemical Society).

Finally, the self-contained, fully integrated lab-on-PCB for sample preparation, PCR amplification, and DNA detection [11] is one of the most representative lab-on-PCB which uses, among other things, electrochemical flow driving. The device includes microchannels, microchambers, micromixers, microvalves, micropumps, and microheaters. The chip is able to manipulate initial samples in the order of microliters and milliliters. In order to do so, electrochemical micropumps are used for driving milliliter volumes. It is based on the electrolysis of water between two platinum electrodes to generate gases when a current is applied. These gases increase the pressure that moves liquids in the device. In addition, a thermopneumatic pump for driving microliter volumes is used. The expansion of the gas is performed in a chamber attached to a PCB-based and resistive microheater. The resulting air expansion drives the liquids into the microchannels and microchambers of the device. Moreover, this device also integrates paraffin-based microvalves for fluid manipulation in lab-on-PCB; see Figure 13. The same authors from Motorola Labs used these actuators for DNA amplification, Ref. [83]. The device was composed of a PCR chamber and paraffin-based microvalves. *Escherichia coli* K12 cells were used in the experiments. Regarding the main fabrication materials, the device was built using mass production materials, that is, polycarbonate and a PCB as the substrate. The fluid manipulation was performed by resistive microheaters to provide the thermal actuation to the paraffin.

Summarizing, the mentioned paraffin-based lab-on-PCBs are robust platforms for microfluidic handling. The reported results are promising, as can be deducted from the reported biomedical applications. Nevertheless, the integration of the paraffin implies complex fabrication steps which go against the low cost mass production, and the subsequent competitiveness as a commercial product. The electrochemical methods are also robust but they have the same drawback as the paraffin-based one, that is, the integration of a small quantities of materials—in this case, water or hydrogels.

## 7. Other Methods

Apart from the previously mentioned methods, there are additional procedures related to flow driving on lab-on-PCBs. These methods have been reported to a lesser extent, but they are worthy of being mentioned. The methods mentioned in this section are peristaltic PCB-based impulsion, passive microfluidics, and several lab-on-PCB compatible methods.

The microfluidic peristaltic pumps are based on microchannels’ deformation to move small volumes of fluid from the inlet towards the outlet, achieving a net continuous flow [84,85]. In order to do so, an electronic AC signal is used for driving the fluid. These micropumps can easily change the direction of the flow. In addition, the flow rate and pressure can be modified by changing the peak-to-peak voltage values, the frequency, and the phase difference of the driving voltages. Nevertheless, they have several drawbacks, such as moving parts and large working area on the substrate.

The integration of peristaltic pumps into printed circuit boards is not easy. This task implies the development of actuators with moving parts to achieve the peristaltic effect. This could be the reason for the lack of pumps of this kind for lab-on-PCBs, if compared with other impulsion methods. However, there are several approaches of PCB-based peristaltic micropumps for lab-on-PCBs. One of these devices was reported in [86,87]. These pumps were composed of a printed circuit board and piezoelectric commercial actuators (buzzers in electronic equipment). The experimental results show a maximum working frequency of 50 Hz with a maximum speed of 3 mm/min for a driven voltage of 140 V. The same working principle was used in the device described in [88]. In this case, the maximum average flow rate was 500 μL/min when the peak-to-peak driven voltage was 100 V at 10 Hz, the maximum backpressure being 760 Pa. The dimensions of the PCB substrate when the pump is integrated were about 10 × 6 cm${}^{2}$. This seems to be large for a integrated pump, but microfluidic circuits and sensors could be included in the remaining area, or even on the opposite side of the PCB.

As a conclusion, it is important to highlight that the main drawback is the required moving parts and its integration into the PCB substrate. That integration implies increasing both the complexity of the fabrication process and the dimensions of the device. Although the PCB substrate makes the device cheaper, the mentioned disadvantages go against the target of lab-on-PCB devices, that is, the inexpensive mass production focused on the market. In fact, up to now, the characteristics of the PCB substrate have not shown interesting advantages in the fabrication of these pumps. Unlike the systems for fluid manipulation mentioned in previous sections, in peristaltic micropumps the copper layer cannot be used for developing the actuator in charge of the liquid movement. In addition, they need external or integrated liquid reservoirs.

The surface acoustic wave (SAW) methods for manipulating fluids are commonly used in microfluidics and lab-on-chip [52,89]. The development of this kind of device using printed circuit boards is not easy due to the substrate material having to be piezoelectric. The typical application of PCBs to these devices is the connection between the radio frequency signal generator and the SAW actuator [90]. However, the microelectrodes can be developed using the copper layer of a PCB substrate. In this case, these electrodes have to be clamped to a piezoelectric substrate. This solution was reported in [91] for fluid and cell manipulations with maximum droplet velocities of about 40 mm/s.

The PCB-based liquid dispenser reported in [92] is a microfluidic pump composed of a oscillating membrane made from a flexible PCB, and magnetically actuated via Lorentz force. Following with the same application, a PCB-based dispenser for biomedical integrates a bending CNT actuator into a PCB, which enables the induction of movement [93]. In addition, a thermally actuated one-shot liquid dispenser, with an actuation based on highly expandable microspheres on PCB has been reported [94].

On the other hand, passive microfluidics is other typical and well-known method used for driving fluids [95]. This method has also been used for printed circuit boards to develop high-performance PCB-based capillary pumps for affordable point-of-care diagnostics [96,97]. The flow can be created without actuation; for example, take the PCB-based self-breathing fuel cells [98,99]. The working itself leads the motion of the liquid. Despite all this, the handling of fluids is one of the most important issues for lab-on-chips and lab-on-PCBs.

There are interesting flow driving methods compatible with lab-on-PCB devices, but they have been developed using different substrates. The first one is based on the integration of thermo-expanding microspheres [100] into silicon substrates. These microspheres were also used for blocking the flow in microchannels. The second one is the impulsion system reported in [101,102] which uses azobis-isobutyronitrile (AIBN) as the solid chemical propellant and a gold microheater. This device is composed of a cyclic oleofin copolymer substrate and gold as a metal. The AIBN is heated at 70 ${}^{\circ }$C to produce nitrogen gas in order to impulse the liquid samples. Finally, dielectrowetting is an interesting compatible technique which creates stronger wetting forces than EWOD [103]. Droplets can be created, transported, splitted, and merged using this technique [104].

## 8. Discussion and Conclusions

The use of the copper layers of the PCB to develop actuators has provided good results. Several PCB-based actuators are easily integrable using that layer, for example, microheaters, microvalves, and electrodes. These actuators have successfully been used for driving flow on lab-on-PCBs. The level of development in the majority of them is suitable. However, the integration of these actuators has drawbacks. The first one is the increase of the area of the substrate due to the integration of the actuator. This fact means an increase of the cost of the final product, especially for single use devices. In this respect, the use of external impulsion devices for lab-on-PCBs implies smaller, simpler and cheaper PCB-chips. This is important because the PCB-chips are the most important source of benefits, especially if they are disposable. In addition, the use of external impulsion devices makes the quality control of the flow driving easy. The reason for this characteristic lies in the use of a unique device for driving fluids for many lab-on-PCBs, instead of a new integrated device for each lab-on-PCBs. Therefore, the use of external impulsion devices is a good choice for no portable lab-on-PCB devices. One drawback of these devices is the change of the tubing for each experiment due to the contamination. This trend could suggest focusing the research on the miniaturization of external devices with no integration so that the portability is applied to the whole system, that is, the electronic control device with small external impulsion devices, and more importantly, small disposable PCB chips. This implies the development of well-defined and standard fluidic and mechanical ports.

It is important to mention that the flow driven devices are not always necessary for lab-on-PCBs. Examples of these devices include the wearable detection biomedical device for real-time detection of glucose in sweat reported in [105] and a PCB-Based thermocycler for PCR [106]. These devices do not require the motion of fluids.

Table 2 summarizes the lab-on-PCB devices with the flow-driving active mechanisms mentioned in this review, except the external energy sources. The table shows the flow driving method and the actuator, the fabrication materials additional to the PCB substrate, the nature of the flow, and the application of the lab-on-PCB. In addition, Table 3 shows a quantitative comparison.

This review described the active flow driving methods for lab-on-PCB devices, mentioning their characteristics from the industrial point of view. In general, there is no perfect method because all of them have drawbacks.

The main problem with regard to marketable devices is the complex fabrication due to the integration of additional fabrication material. In addition, the method of assembly of the LoPs increases that difficulty.

Moreover, the biomedical and biochemical applications are very demanding. Among other things, they require transparency, biocompatibility, and specific ambient conditions, for example, temperature, humidity, or pH. Particularly, these requirements have to be compatible with the flow driving method, together with the rest of the lab-on-PCB, including the sensing. All the problems have to be solved to provide consistency and reliability for both the fabrication process and the function. In this respect, the most developed method for commercial PCB-based flow driving applications, which better fulfills those requirements, is electrowetting on dielectrics. Nevertheless, it has drawbacks which could be solved with the combination of EWOD with a different flow driving method.

As can be seen in this review, lab-on-PCB devices have been developed for many biomedical and biochemical applications. However, much work has to be done towards commercial applications. This is especially challenging if taking into account the boundary conditions of the market, that is, repeatability, reliability, and acceptable fabrication processes. Even so, the research of devices of this kind is rapidly increasing, so interesting solutions are being developed day to day. The reason for this lies in the great potential of lab-on-PCB devices to provide marketable devices.

## Figures and Tables

**Table 1:** Main differentiating characteristics of lab-on-chip and lab-on-PCB.

Characteristic	Lab-on-Chip	Lab-on-PCB
Materials for microfluidics	Silicon, glass, plastic	PCB, plastic
Substrate materials	Silicon, glass, plastic	PCB (rigid/flexible)
Maximum number of metal layers	2 (except silicon)	30 [15]
Fabrication of electronic tracks	Yes	Yes (low cost)
Commercially available substrate	Yes	Yes (low cost/integrated electronics)
Impact-resistant chips	Brittle (Silicon, glass)	Robust
Transparency	Yes	No
Highly integrated electronics	Yes (Silicon)	No
Discrete electronic components	SMD	SMD and through hole
Sensors/actuators integration	Yes	Yes (low cost)
Sensing performance	High	Medium
Biocompatibility	Yes	Yes (insulation layer)
Best application scenario	Optical and/or high sensitivity	The rest of the applications
Disposable at low cost	Yes (plastic)	Yes
Potential of commercialization	Low	Very high

**Table 2:** Characteristics of active microfluidic, pressure-driven lab-on-PCBs.

Method	Actuator	Materials	Flow	Application	References
Pressurized	valve	SU-8	Samples	General Purpose	[40,44,46,48]
Pressurized	valve	SU-8/Au	Samples	General Purpose	[42,47]
Pressurized	valve	PMMA	Samples	General Purpose	[49]
EWOD	Electrodes	Dielectric	Droplet	General Purpose	[53,55,56,62]
EWOD	Electrodes	Glass/dielectric	Droplet	General Purpose	[54,59,60,61]
EWOD	Electrodes	Polymer/dielectric	Droplet	Clinical diagnosis	[64]
EWOD	Electrodes	SU-8/Teflon	Droplet	General Purpose	[58]
Osmotic	Electrodes	SU-8	Continuous	General Purpose	[69,70]
Osmotic	Electrodes	1002F	Continuous	General Purpose	[71]
Osmotic	Electrodes	Gold	Continuous	General Purpose	[72]
Osmotic	Electrodes	AAO/PDMS	Continuous	General Purpose	[73]
Peristaltic	Piezo-disc	Brass	Continuous	General Purpose	[86,87,88]
Paraffin	Heater	Paraffin/Epoxy	Samples	General Purpose	[76]
Paraffin	Heater	Paraffin/PDMS	Samples	DNA extraction	[77]
Thermoneumatic	Heater	Paraffin/PC	Samples	DNA amplification	[11]
Electrolytic	Heater	Paraffin/PC	Samples	DNA amplification	[11]
Electrolytic	Electrodes	PMMA	Continuous	General Purpose	[78,79]
Electrolytic	Electrodes	Polymer/Pt	Continuous	General Purpose	[80]
Electrolytic	Electrodes	Polymer	Continuous	PCR and Analysis	[81]
Electrolytic	Electrodes	Glass	Continuous	Droplet generation	[82]
SAW	Electrodes	$LiNbO_{3}$	Droplet	Cell/droplet manipulation	[91]
Magnetic	Magnet	NdFeB	Sample	Dispenser	[92]
CNT-based	CNT-actuator	CNT	Sample	Dispenser	[93]
Microspheres	Heater	Polymer	Sample	Dispenser	[94]

**Table 3:** Lab-on-PCB devices and flow driving: a quantitative comparison.

Method	Module	Fabrication	Fluidic	Channel	Reference
	Dimension	Complexity/Cost	Condition	Section	
Pressurized	3 × 2 cm${}^{2}$	Medium/Low	Average speed	0.3 × 1 mm ${}^{2}$	[40]
chamber			41 mm/s		
Pressurized	7.5 × 4.5 cm${}^{2}$	Medium/Low	Average speed	0.3 × 0.5 mm ${}^{2}$	[48]
chamber			16.25 mm/s		
Pressurized	6 × 1 cm${}^{2}$	High/High	Average speed	0.35 × 0.5 mm ${}^{2}$	[47]
chamber			271 mm/s		
Pressurized	6 × 5 cm${}^{2}$	Low /Low	Average flow rate	1 × 1.1 mm ${}^{2}$	[49]
chamber			1.4 μL/s		
EWOD	8.9 × 8.9 cm${}^{2}$	Low /Low	Max speed	No channel	[53]
	1024 electrodes		100 mm/s		
EWOD	∼14 × 10 cm${}^{2}$	Low /Low	Max speed	No channel	[56]
	6 electrodes		3 mm/s		
EWOD	N/A	Low/Low	Velocity	No channel	[61]
	64 electrodes		6–13 mm/s		
EWOD	12.78 × 8.55 cm${}^{2}$	Medium/Low	N/A	No channel	[63,64]
	N/A electrodes				
EWOD	5 × 5 cm${}^{2}$	High/Low	N/A	No channel	[58]
	576 electrodes				
Osmotic	∼4.5 × 4.5 cm${}^{2}$	Medium/Low	Flow rate	0.2 × 0.1 mm ${}^{2}$	[69,70]
	6 pumps		1 μL/min		
Osmotic	7.62 × 2.54 cm${}^{2}$	High/Low	Max speed	0.3 × 0.07 mm ${}^{2}$	[71]
	1 pump		800 μm/s		
Osmotic	2 × 1 cm${}^{2}$	Low/High	Max speed	N/A	[72]
	1 pump		750 μm/s		
Osmotic	15 × 15 cm${}^{2}$	High/Medium	Flow rate	Nanopore	[73]
	1 pump		8 μL/min	Membrane	
Peristaltic	∼10 × 6 cm${}^{2}$	Low/Low	Average flow rate	N/A	[88]
	1 pump		500 μL/min		
Peristaltic	∼0.4 × 0.25 cm${}^{2}$	Low/Low	Max flow rate	N/A	[86,87]
	1 pump		1500 μL/min		
Paraffin	5.5 × 4 cm${}^{2}$	High/Low	Max flow rate	N/A	[76]
			240 μL/min		
Thermoneumatic	10 × 6 cm${}^{2}$	High/Low	Moved volumen	Min	[11]
			60 μL	0.3 × 1 mm ${}^{2}$	
Electrolytic	10 × 6 cm${}^{2}$	High /Low	Max flow rate	Min	[11]
			0.8 μL/min	0.3 × 1 mm ${}^{2}$	
Electrolytic	10 × 6.5 cm${}^{2}$	Medium/High	Flow rate-backpressure	1.5 × 2.5 mm	[78,79]
			31.6 mL/min–547 kPa		
Electrolytic	2.5 × 1 cm${}^{2}$	Low/Low	Max flow rate	1 × 0.025 mm	[80]
			135 μL/min		
Electrolytic	8 × 6 cm${}^{2}$	High/Low	Max flow rate	N/A	[81]
			0.1–1 μL/s		
Electrolytic	N/A	Medium/Low	Max flow rate	0.15 × 0.06 mm	[82]
			100 μL/min		
SAW	11 × 11 cm	Low/Medium	Max speed	0.15 × 0.06 mm	[91]
			40 mm/s		
Magnetic	∼4 × 3 cm	Low/Low	N/A	1 × 1 mm	[93]

## Data Availability

Data is contained within the article.

## Abbreviations

The following abbreviations are used in this manuscript:

MDPI	Multidisciplinary Digital Publishing Institute
DOAJ	Directory of open access journals
TLA	Three letter acronym
LD	linear dichroism
